# Cap assisted colonoscopy for the detection of serrated polyps: a post-hoc analysis

**DOI:** 10.1186/s12876-015-0234-1

**Published:** 2015-02-05

**Authors:** Fadi Rzouq, Neil Gupta, Sachin Wani, Prateek Sharma, Ajay Bansal, Amit Rastogi

**Affiliations:** 1 Department of Internal Medicine, Division of Gastroenterology and Hepatology, University of Kansas School of Medicine, Kansas City, KS 66160 USA; 2 Department of Gastroenterology, Loyola University Medical Center, Maywood, IL 60153 USA; 3 Department of Gastroenterology, University of Colorado and Veterans Affairs Medical Center, Aurora, CO 80045 USA; 4 Department of Gastroenterology, University of Kansas School of Medicine, Kansas City Veterans Affairs Medical Center, Kansas City, MO 64128 USA

**Keywords:** Colorectal cancer, Serrated polyps, Cap-assisted colonoscopy, Interval colon cancer

## Abstract

**Background:**

Colonoscopy offers limited protection against right-sided colon cancer, a significant proportion of which arise from the serrated pathway of carcinogenesis. The aim of this study was to compare cap-assisted colonoscopy and standard high-definition white light colonoscopy regarding serrated polyps’ detection.

**Methods:**

Post hoc analysis was performed of a previously conducted randomized controlled trial comparing standard and cap-assisted colonoscopy for adenoma detection. Randomization was stratified based on the indication of colonoscopy and all procedures were performed by three experienced endoscopists. Following cecal intubation, the colonic mucosa was carefully inspected during withdrawal of colonoscope and all polyps detected were documented for their size, location, morphology and then removed and sent for histopathology. Detection rates of significant serrated polyps between both arms were compared using the Fisher’s exact test and Wilcoxon Rank Sum test.

**Results:**

427 patients were enrolled (7 exclusions, 210 completed study in each arm, mean age of 61 years, 95% male, 75% Caucasian, 67% screening colonoscopies). There were no significant differences in baseline characteristics between both groups. Cap-assisted colonoscopy detected a significantly higher proportion of subjects with significant serrated polyps as well as a higher total number of significant serrated polyps compared to standard colonoscopy (12.8% vs. 6.6%, p =0.047 and 40 vs. 20,p = 0.03 respectively).

**Conclusions:**

In this post-hoc analysis, Cap-assisted colonoscopy is a safe technique that offers a higher detection rate of significant serrated polyps when compared to standard colonoscopy. If confirmed in future trials, this simple technique has the potential to improve protection against interval colon cancers.

## Background

Colonoscopy is one of the recommended screening tests for colorectal cancer (CRC) in the US. The added benefit of colonoscopy in preventing CRC makes it the preferred modality compared to the other screening tests [1]. The basic mechanism by which colonoscopy prevents CRC is by the detection and removal of adenomas that are the precursor lesions for cancer [2]. However, the magnitude of CRC protection has been shown in multiple studies to be site dependent (proximal vs distal colon) with proximal (right sided) CRC protection being less pronounced [3,4]. The factors accounting for this lower protection against proximal CRC have been a matter of debate with multiple potential explanations put forth by investigators. One such explanation is the presence of serrated lesions in the right colon and the inability to detect them during colonoscopy resulting in a higher incidence of interval colon cancer in the proximal colon [5] through the serrated pathway of colorectal carcinogenesis.

As per the latest WHO classification, serrated polyps are classified into three categories; hyperplastic polyps, sessile serrated adenoma/polyps, and traditional serrated adenomas [6]. These polyps possess variable endoscopic and histopathologic features [7] as well as variable malignant potential [8]. These lesions are subtle and non polypoid (flat) making them susceptible to being missed during colonoscopy [9]. Studies have shown that there is a wide variation in the detection of proximal serrated polyps between endoscopists which alludes to the fact that these polyps are being missed during colonoscopy and maybe even more so than adenomas [10] and also associated with higher rates of incomplete resection [11]. Therefore improving proximal serrated polyp detection rate is being recognized as an important goal that may improve the protection afforded by colonoscopy against right sided colon cancer [12].

Multiple strategies have been evaluated to improve the polyp/adenoma detection rate during colonoscopy [13]. Cap assisted colonoscopy (CAC) is one such technique that has been investigated and shown to be safe with some benefit in improving the polyp/adenoma detection rates [14]. However, CAC has not been well studied for the detection of serrated polyps although a recent randomized controlled study comparing adenoma detection between conventional colonoscopy and CAC has provided a subgroup analysis for the detection of serrated polyps with no difference between the two arms [15]. The aim of this study was to compare the detection of significant serrated polyps [sessile serrated adenoma/polyp, traditional serrated adenoma, proximal hyperplastic polyps and large (≥1 cm) hyperplastic polyp] between CAC and standard colonoscopy.

## Methods

### Study design

We conducted a post hoc analysis of a previously conducted randomized controlled trial comparing standard (SC) and cap assisted colonoscopy (CAC) for adenoma detection [14]. The original study was a prospective randomized controlled trial conducted at the Kansas City Veterans Affairs Medical Center and was approved by its Institutional Review Board (KCVA IRB) and was registered at http://clinicaltrial.gov (NCT 01211132). The primary results have been previously reported [16].

### Study subjects

Subjects who were referred for screening or surveillance colonoscopy were prospectively enrolled between September 2009 and October 2010. Subjects were included in the study if they were referred for a screening or surveillance colonoscopy and agreed to provide a written informed consent. The procedure was considered a screening one if the patient never had a colonoscopy before or if no polyps were detected on previous colonoscopies. On the other hand, subjects with a history of adenoma on a prior colonoscopy and who were referred for follow-up were deemed to be undergoing surveillance colonoscopy.

The exclusion criteria of the study included previous surgical resection of any part of the colon, personal history of colon cancer, history of inflammatory bowel disease, use of antiplatelet agents or anti-coagulants that precluded removal of polyps, poor general condition or any other reason to avoid prolonged procedure time, history of polyposis syndrome or hereditary nonpolyposis colorectal cancer, or the inability to give an informed consent.

Following enrollment and randomization to one of the arms, those subjects who had inadequate colon preparation or in whom the cecum could not be reached were also excluded. Different patient parameters were recorded included demographics (age, gender and race), indication of colonoscopy, body mass index (BMI), family history of colorectal cancer (first-degree relative), history of smoking, and history of prior abdominal surgery.

### Randomization

Following enrollment, subjects were randomized to undergo either SC or CAC by opening an opaque sealed envelope containing the procedure allocation. The randomization sequence was stratified by the indication of colonoscopy (screening vs. surveillance) and was generated by the statistician with a computerized randomization scheme using block sizes of 10.

### Colonoscopy procedure

Moderate sedation was used in the study using intravenous midazolam in combination with either meperidine or fentanyl. The colonoscope was inserted and advanced to the cecum. Careful mucosal inspection and polypectomies were performed during the withdrawal phase. Procedural times were recorded using a stopwatch (the cecal intubation time as well as withdrawal time). The stopwatch was not paused during any maneuver intended to assist cecal intubation (like suctioning of fluid, cleaning of the mucosa, abdominal pressure, or changing of position). The cecum was identified using the landmarks including the appendiceal orifice (which was photo documented) and intubation of the terminal ileum was attempted in all cases and its successful intubation was recorded. The withdrawal time in the study was defined as the time spent on mucosal inspection while the colonoscope was being withdrawn. The stopwatch was paused whenever the colonic mucosa was not being examined (for example during suctioning of fluid or during biopsies and polypectomy). In the CAC procedures, the cap was used to flatten the mucosal folds in order to inspect their proximal aspect for polyps.

Bowel preparation was evaluated and was then classified into four categories [17] as follows: excellent (>90% of mucosa seen, the colonic contents were mostly liquid with minimal suctioning needed for clearing the mucosa), good (same as excellent except that significant suctioning was needed for clearing the mucosa), fair same as excellent except that the colonic contents were a mixture of liquid and semisolid which could be suctioned and/or washed), and inadequate (<90% of mucosa seen, the colonic contents were a mixture of semisolid and solid colonic contents, which could not be suctioned or washed). All procedural complications were recorded (like bleeding or perforation). Endoscopists were asked to document any technical difficult encountered with the cap like cap dislodgement or any difficulty in suctioning fluid or cleaning the mucosa or during therapeutic interventions attributable to the cap.

### Study endpoints and statistical analysis

All analyses were performed using Stata/IC V.10.1 (StataCorp). The original study was powered to the primary end point that was the proportion of subjects with at least one adenoma. Fisher’s exact test, *t* test, and Wilcoxon rank sum test were used to compare the differences across the two study groups regarding the total number of subjects with significant serrated polyps as well as the total number of significant serrated polyps detected. Similar comparisons were made for the individual categories of serrated polyps. A p value <0.05 was considered statistically significant and the results were reported in accordance to the guidelines of CONSORT 2010 [18] and Standards for Reporting of Diagnostic Accuracy [19].

### Definitions

Significant serrated polyps were defined in this study as sessile serrated adenoma/polyp, traditional serrated adenoma, proximal hyperplastic polyps and large (≥1 cm) hyperplastic polyp.

### Pathology review

All specimens were reviewed by an expert GI-pathologist who is well-familiar with the recent WHO classification for serrated polyposis.

## Results

### Subjects and procedural characteristics

A total of 427 subjects were prospectively enrolled by 3 endoscopists in this randomized controlled trial between September 2009 and October 2010. Seven subjects were excluded (five due to failed cecal intubation, one due to inadequate bowel preparation, and one due to the diagnosis of Crohn’s disease). Thus, a total of 420 patients (210 in each of the two arms) completed the study protocol (mean age of 61 years, 95% male, 75% Caucasian, 67% screening colonoscopies, 33% surveillance colonoscopies). Baseline characteristics of subjects in both study arms were similar like age, gender, race, smoking history, BMI, and family history of colon cancer. Successful cecal intubation was seen in the vast majority of cases (98% of procedures in the SC group as compared to 99% in the CAC group with p = 0.37). The mean insertion time was significantly shorter in the CAC group as compared to the SC group (3.29 +/− 2.55 min vs. 3.98 +/− 2.56 min, p < 0.001) and the terminal ileal intubation rate was higher in the CAC group (91% vs. 83%, p 0.018). On the other hand, the mean withdrawal time (5.95 ± 1.58 min in CAC vs 5.98 ± 1.45 min for standard colonoscopy, p = 0.75), bowel preparation quality, and sedation medication doses were not significantly different between the two groups.

### Serrated polyps detection rate

As outlined in Table 1, CAC detected higher total number of significant serrated polyps when compared to SC (40 vs 20; P 0.032). For subgroup analysis, CAC detected a higher number of SSA/P or TSA (7 vs 1, p 0.03). Although the number of proximal hyperplastic polyps (26 vs 14; P 0.1) and large hyperplastic polyps (12 vs 7; P 0.19) was numerically higher with CAC, the differences did not achieve statistical significance.

**Table 1:** **Significant serrated polyps’ detection**

	SC	CAC	P value
	N = 210	N = 210	
Total Number of Significant Serrated Polyp	20	40	0.032
Total Number of Proximal Hyperplastic Polyp	14	26	0.10
Total Number of Large Hyperplastic Polyps	7	12	0.19
Total Number of SSA or TSA	1	7	0.03

Similarly, the proportion of subjects with significant serrated polyps was higher in the CAC as compared to the SC group (13% vs 7%; P 0.047). For the subgroup analysis, the proportion of subjects with SSA/SSP or TSA, proximal HP, large HP, were numerically higher in the CAC compared to SC but these differences did not reach statistical significance for any subgroup (Table 2).

**Table 2:** **Proportion of subjects with significant serrated polyps**

	SC	CAC	P value
	N = 210	N = 210	
Significant Serrated Polyp (%)	14 (7%)	27 (13%)	0.047
Proximal Hyperplastic Polyp (%)	12 (6%)	21 (10%)	0.15
Large Hyperplastic Polyps (%)	5 (2%)	10 (5%)	0.29
SSA or TSA (%)	1 (0.5%)	7 (3%)	0.07

## Discussion

Our knowledge about serrated polyps continues to evolve. The recent WHO classification divided them into 3 categories: hyperplastic polyps, sessile serrated adenoma/polyps, and traditional serrated adenomas [6]. While previously all hyperplastic polyps were considered to have no malignant potential [20], there has been a recent shift in the understanding. Expert consensus has deemed proximal hyperplastic polyps to be of some neoplastic potential contrary to what was previously thought [21]. Moreover, serrated polyps in the proximal colon may have been misclassified as hyperplastic subtype by the pathologists to begin with [22]. Along with this multiple studies have shown that there is marked variability amongst the pathologists in differentiating between hyperplastic polyps and SSA/P [23,24]. As a result in this study we included proximal and large hyperplastic polyps along with SSA/P and TSA in a broad category of significant serrated polyps recognizing their malignant potential.

CAC can serve as a simple technique to help examine the mucosa on the proximal aspects of colonic. After application on to the tip of the colonoscope, the cap protrudes for about 4 mm. This portion helps to flatten the colonic folds and brings the mucosa on their proximal aspects in the view of the endoscopist. In the primary results of this study reported earlier, we showed that CAC significantly improved the adenoma detection rates [16] while other studies have showed varying results with CAC [14].

We herein report the results of a post hoc analysis of a previously published randomized controlled trial comparing CAC and standard colonoscopy [16]. We found that CAC showed a significantly higher detection rate for significant serrated polyps and also for the proportion of subjects who had at least one significant serrated polyp. The comparisons for the subtypes of serrated polyps did not show a significant difference except for the total number of SSA/TSA that was significantly higher for the CAC group. The other subcategories were numerically higher for CAC and the lack of statistical significance could very well be explained by a Type II error, as the trial was not powered adequately to detect a difference in these categories. Future randomized controlled trials specifically powered to detect a difference in the detection of serrated polyps between CAC and standard colonoscopy are warranted. Moreover, whether this improved detection of serrated polyps by CAC has any positive impact on the future development of proximal CRC will need further assessment by longer term follow up studies.

The major appeal of CAC lies in the fact that it is a relatively inexpensive technology that is easy to use and safe. Other methods to examine the proximal aspect of folds involve third eye retroscope [25], retroflexion of the colonoscope in the right colon [26] and recently available wide angle colonoscopes [27]. The third eye retroscope is expensive and relatively more cumbersome to use as it needs to be removed after a polyp is detected and then reinserted after polypectomy. Retroflexion in the right colon is not widely practiced and in one study, a second exam of the proximal colon in retroflexion did not show superior polyp detection compared to a second standard exam [28]. The extra wide angle colonoscope that has a 330 degree angle of view has just recently become available. Although preliminary studies have been encouraging [29], more data from multiple centers is awaited. It remains to say that other inexpensive and safe technologies exist to examine behind folds like the endocuff. The importance of evaluating and implementing new strategies to improve the detection of serrated polyps is related to the fact that this group of polyps has a rather subtle appearance and non-polypoid morphology making them susceptible to be missed during colonoscopy. This was shown in a recent study in which there was a very wide variability in the detection rates for proximal serrated polyps amongst endoscopists practicing in a single institution [10]. Furthermore, due to their subtle appearance, serrated polyps are also more likely to be incompletely resected compared to adenomatous polyps of same size, location, and morphology [11]. This can also have implications for the development of interval colon cancer as a result of incomplete polypectomy.

The major strength of this study relies in the fact that serrated polyposis is a significant source for colorectal cancer and CAC is a safe and cheap technique that may significantly improve the detection of these polyps and help boost colonoscopy protection against colorectal cancer development.

However, our study has several limitations. First, the study was performed in an academic setting by three experienced endoscopists and thus it remains unclear if these results can be generalized to the community endoscopists. Second, the original study was not powered to detect a difference in the detection of serrated polyps. This raises the possibility of a Type II error as an explanation for the lack of statistical significance in the differences seen in the detection of the serrated polyps subtypes between the two arms. Third, the majority of patients enrolled in the study were male veterans and Caucasians and the results may not be generalizable to the general population. Fourth, as a consequence of the study design (post-hoc analysis), the distal small hyperplastic polyps were not removed during colonoscopy. Fifth, our mean withdrawal time was less than 6 minutes for both groups (although was not different between the two groups). Finally, the presence of investigator bias remains as issue in endoscopic studies such as this one since the endoscopists cannot be blinded to the technology that is being compared to the standard colonoscopy.

## Conclusions

In conclusion, we have shown by a post hoc analysis of a randomized controlled trial that CAC detects a higher total number of significant serrated polyps as well as higher number of patients with significant serrated polyps compared to standard colonoscopy. CAC is a safe, practical and easy to use method to improve the detection of these clinically significant polyps. Further studies specifically powered and aimed to compare the detection of the specific sub types of serrated polyps between CAC and standard colonoscopy are warranted.
